# An Unusual Case of Subacute Appendicitis and Intestinal Spirochetosis

**DOI:** 10.7759/cureus.18050

**Published:** 2021-09-17

**Authors:** Eman Chaudhri, Abdul Hakim Almakadma, Sami Almustanyir, Abdulaziz Taleb, Nawaf Alotaibi

**Affiliations:** 1 Medicine, Alfaisal University, Riyadh, SAU; 2 Internal Medicine, Ministry of Health, Riyadh, SAU; 3 Gastroenterology, King Fahad Medical City, Riyadh, SAU

**Keywords:** intestinal spirochetosis, appendicitis, gastrointestinal infection, rlq pain, irritable bowel disease

## Abstract

Intestinal spirochetosis is a gastrointestinal infection with vague and inconsistent symptoms. It similarly presents multiple gastrointestinal diseases such as inflammatory bowel disease and appendicitis. We present a case of a 27-year-old female with intestinal spirochetosis who was later found to have subacute appendicitis. Further understanding of the disease and a set of criteria may have to be created for its management.

## Introduction

Intestinal spirochetosis (IS) is a gastrointestinal infection with an increasing global prevalence. However, the underlying pathophysiology of spirochetosis is not fully understood. Spirochetes are found in the digestive tracts of many species, including humans [1]. Additionally, the clinical presentation of the infection is usually vague and can be similar to other gastrointestinal infections and conditions, such as appendicitis, irritable bowel syndrome and inflammatory bowel disease [2]. The vague nature and presentation of IS highlight the clinical importance of recognizing the disease.

## Case presentation

A 27-year-old female presented with gradually progressive severe abdominal pain for four months. The pain has been radiating to the right lower quadrant (RLQ) and recently extended to the right groin. The pain was associated with minimal guarding and rebound tenderness. She also had nausea, vomiting and mild diarrhea. Furthermore, she reported 3-4 kg weight loss in the past two months. There is no known previous medical or family history. The patient was afebrile and vitals were stable. Initial laboratory findings provided in Table 1 were insignificant.

**Table 1:** Initial laboratory markers WBC: White Blood Cells; CRP: C-Reactive Protein

Laboratory Parameters	Value	Normal Range
Hemoglobin	13.2	12-16 g/dL
WBC	10.02	4.5-11 K/µL
Creatinine	39	53-106 µmol/L
Potassium	4.04	3.6-5.2 mmol/L
Chloride	104	96-106 mEq/L
CO_2_	15.9	23-29 mEq/L
CRP	4	<3.0 mg/L

**Figure 1:**
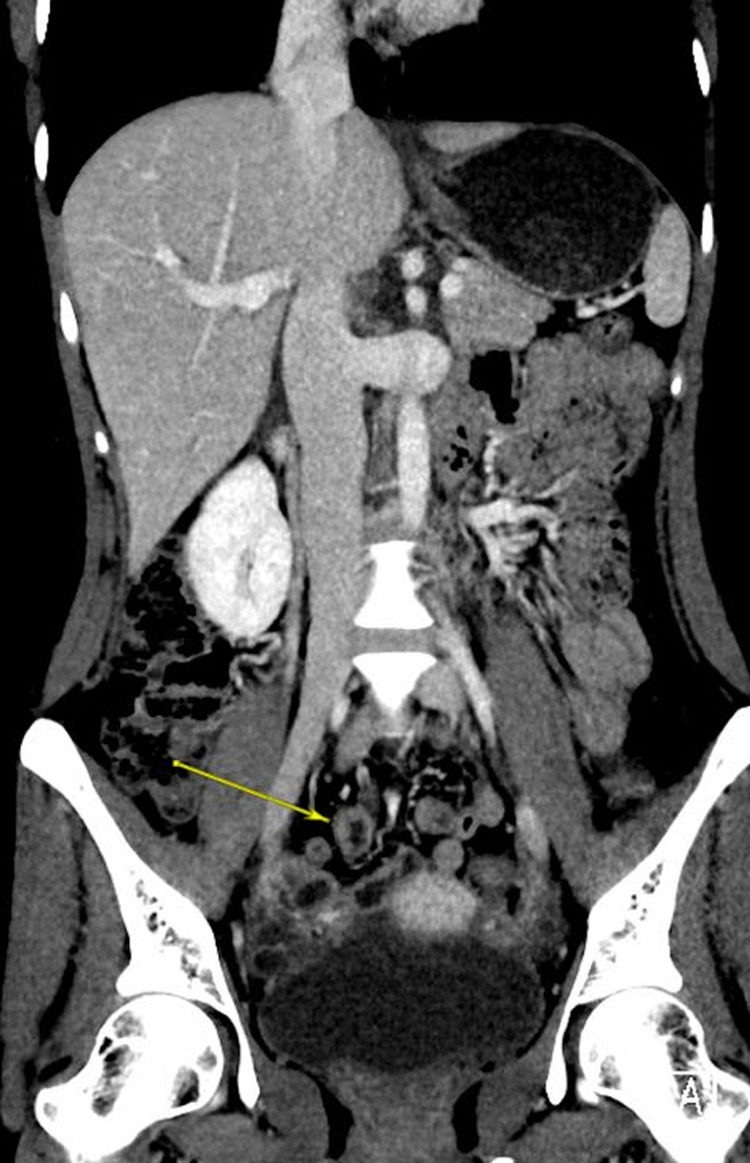
Initial abdominal CT showing multiple segmental thickening of the small bowel wall (yellow arrow)

**Figure 2:**
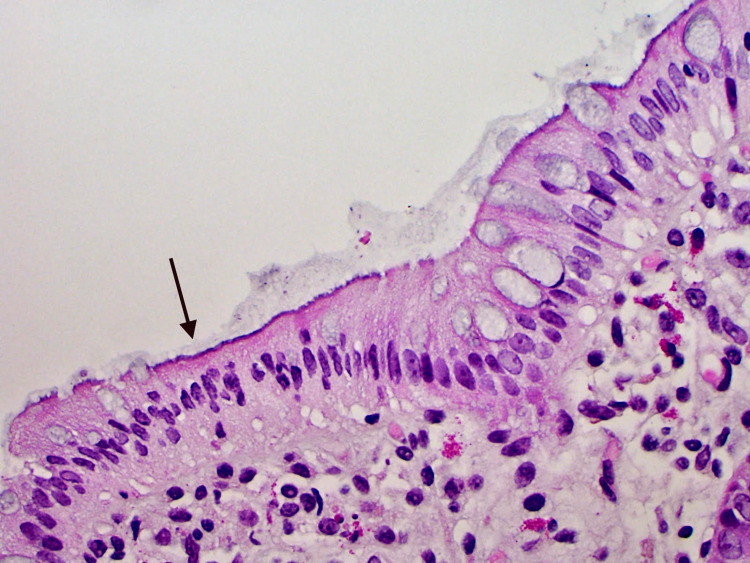
Colonic mucosa biopsy - a periodic acid–Schiff stain showing filamentous structures (black arrow) on the surface epithelium forming a thick bluish fringe

An initial computed tomography (CT) scan provided in Figure 1 showed multiple segmental thickening of the terminal ileum and distal ileal loops with surrounding inflammatory changes. Moreover, it was associated with reactive inflammation of the appendix, most likely secondary to inflammatory/infectious processes. An esophagogastroduodenoscopy (EGD) was performed and was insignificant. A colonoscopy was then conducted, and a biopsy of the colonic mucosa was acquired, which revealed a basophilic fringe-like mildly thickened brush border, suggestive of spirochetosis as illustrated in Figure 2. Periodic acid-Schiff (PAS) and Silver stain showed small filamentous structures on the mucosal surface suggestive for IS. The patient was discharged on Metronidazole for two weeks and the pain subsided.

**Figure 3:**
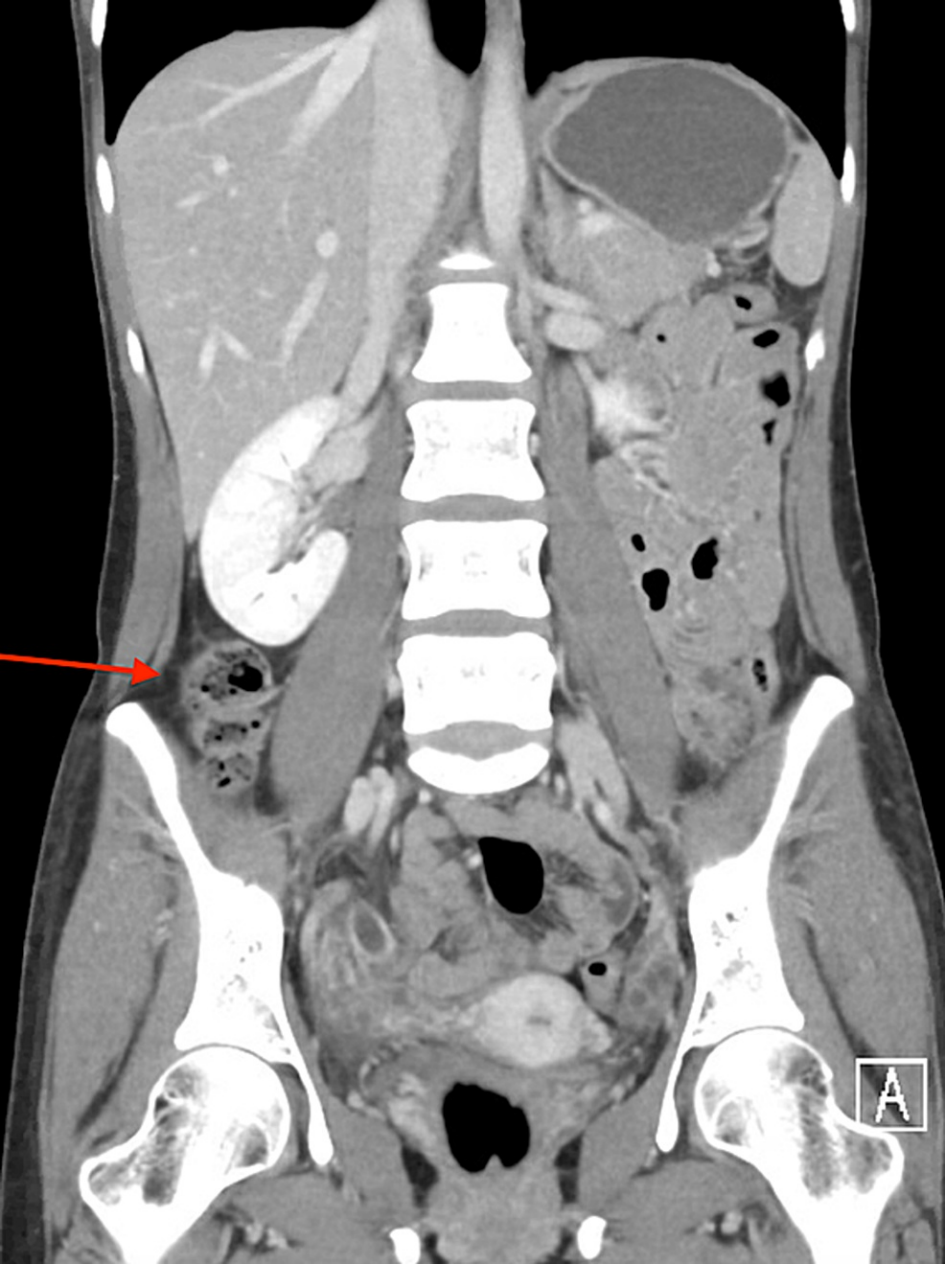
CT abdomen pelvis with contrast showing a dilated appendix (red arrow)

A few days after completion of the Metronidazole course, the patient’s RLQ pain returned. On re-examination, the abdomen was soft, with minimal guarding but positive for rebound tenderness in the RLQ. Repeat laboratory findings are shown in Table 2. Repeat EGD and colonoscopy were both normal. A second CT provided in Figure 3 revealed a dilated appendix with a thick hyper-enhancing wall.

**Table 2:** Repeat laboratory parameters WBC: White Blood Cells; CRP: C-Reactive Protein; ESR: Erythrocyte Sedimentation Rate; HIV: Human Immunodeficiency Virus

Laboratory Parameters	Value	Normal Range
Hemoglobin	11.6	12-16 g/dL
WBC	6.29	4.5-11 K/μL
Fecal Calprotectin	<22.0	10-50 μg/mg
CRP	0.5	<3 mg/L
ESR	30	0-20 mm/hr
HIV	Negative	-

A diagnosis of subacute appendicitis was made. The underlying IS was likely to be benign and not responsible for the patient’s initial presentation. It was agreed on by the general surgery team that management will be conservative and that the patient will be clinically reassessed for surgery upon follow-up. Three weeks later, there was no improvement in the RLQ pain. The patient was accepted for a laparoscopic appendectomy which was performed with no complications. The patient was discharged on post-operation day 1 and was advised to discontinue antibiotics, take analgesics as needed, and follow up in two weeks’ time.

## Discussion

There are three phylogenetic groups of the Spirochaetaceae family. Of which, the Brachyspiraceae are fastidious anaerobic organisms, spread via the fecal-oral route, commonly found in IS [3]. Most cases tend to be asymptomatic; however, patients can present with chronic watery diarrhea, abdominal pain, and occasionally, hematochezia. It is hypothesized that symptoms and clinical presentation are secondary to immune reactions elicited by penetration of spirochetes into the mucosal cells, and by macrophage uptake [4]. However, in many cases, the clinical presentation is vague and unrelated to the presence of intestinal spirochetes and therefore requires further studies.

IS tends to the apical membrane of the colonic epithelium [5]. Therefore, diagnosis is made through histopathologic examination of a mucosal biopsy showing adhesion of spirochetes to the brush border mucosa. IS has been reported more commonly in developing countries, suggesting that diet and sanitation are possible factors contributing to the pathogenesis of the infection [5-7]. There is a prevalence of around 1.1%-5% of IS in developing countries [7]. Further prevalence includes 32.6% in Australian aboriginal people, 64.3% in villages in India amongst otherwise healthy individuals, and 11.4%-26.7% in hospitalized and healthy people, respectively, in Oman.

Spirochetosis has been previously suggested to occur in immunocompromised individuals infected with HIV; however, spirochetosis’s prevalence is increasing in the general population [6]. IS masked by systemic or local bowel disease has been previously discussed [2]. Our patient presented with atypical symptoms of IS, masking the diagnosis of appendicitis. A similar presentation has previously been described in a 13-year-old boy, with a differential diagnosis of IBD or infectious colitis and was then later found to have IS [5].

The diagnosis of IS is challenging and may mask other diagnoses. Only two cases of appendiceal spirochetosis and two cases of colorectal spirochetosis have been discussed in Saudi Arabia [4,8,9]. Our patient had unspecific gastrointestinal symptoms indicating an ongoing inflammatory process; however, her laboratory results were unremarkable and were found to have subacute appendicitis. IS is also commonly found with concurrent gastrointestinal infections, usually involving enteric pathogens such as Helicobacter pylori, Shigella flexneri, and Enterobius vermicularis. Furthermore, IS can be an incidental finding in asymptomatic individuals [3,6]. Moreover, diagnostic procedures are often inconclusive. Colonoscopy may aid in the diagnosis, but findings are non-specific to IS, such as erythematous lesions, polypoid lesions, or normal findings in the majority of the mucosa, as seen in our patient [2]. Histopathology can vary from patient to patient, although a “fuzzy brush border” appearance seems to be the common characteristic [2]. Treatment depends on the presentation and severity of symptoms as well as any other underlying conditions or factors [1]. Antibiotic treatment is indicated in symptomatic cases, and metronidazole seems to be the preferred medication [1,6].

## Conclusions

This case is a novel example suggesting the lack of knowledge regarding the clinical significance of IS. The pathogenesis and pathophysiology of this infection remain unclear and poorly understood. Therefore, a set of guidelines or criteria may have to be put in place to provide clarity for IS diagnosis and treatment. Due to the numerous different clinical presentations of patients with an IS infection, physicians must be made aware of the different presentations. Understanding patients’ immune and socioeconomic statuses may aid in gathering more data and understanding IS better in the future.
